# Methemoglobinemia suspected of being caused by excessive intake of leafy vegetables due to an unbalanced diet: A case report

**DOI:** 10.1097/MD.0000000000046782

**Published:** 2025-12-19

**Authors:** Katsuki Kono, Junko Yamaguchi, Akihiro Noda, Kosaku Kinoshita

**Affiliations:** a Division of Emergency and Critical Care Medicine, Department of Acute Medicine, Nihon University School of Medicine, Tokyo, Japan.

**Keywords:** dietary imbalance, food-induced, leafy vegetables, methemoglobinemia

## Abstract

**Rationale:**

Methemoglobinemia is commonly caused by acute toxic exposure. However, excessive consumption of certain foods may be a contributing factor. Although diet-induced methemoglobinemia has been reported in infants and young children, clinically significant cases suspected of being caused by an unbalanced diet in adults are rare.

**Patient concerns:**

A 60-year-old female presented with persistent dizziness and difficulty walking following a fall.

**Diagnoses:**

Arterial blood gas analysis revealed a methemoglobin level of 25%. Plain radiography confirmed a femoral fracture. A detailed medical history revealed an excessive intake of leafy green vegetables, leading to the diagnosis of diet-induced methemoglobinemia as the underlying cause.

**Interventions:**

The patient received conservative treatment, including normobaric oxygen therapy, which resulted in a decrease in methemoglobin levels within 16 hours of admission.

**Outcomes:**

The patient was discharged on the 6th day of hospitalization and was transferred to another facility for the treatment of the left femoral neck fracture.

**Lessons:**

Excessive intake of leafy vegetables may induce methemoglobinemia and warrants careful consumption.

## 1. Introduction

Methemoglobinemia is a potentially life-threatening disorder characterized by the oxidation of the iron moiety in hemoglobin from the ferrous (Fe²⁺) to the ferric (Fe³⁺) state. Ferric hemoglobin, also known as methemoglobin, is incapable of binding and transporting oxygen. Its presence affects the oxygen-carrying capacity of the blood and shifts the oxygen-hemoglobin dissociation curve to the left, impairing oxygen release to peripheral tissues. As a result, methemoglobinemia leads to functional anemia and tissue hypoxia, even when the partial pressure of arterial oxygen is normal.^[1]^

Methemoglobinemia is most commonly caused by acute toxicity or overdose; however, in some cases, it may be induced by food.^[2]^

Approximately 25% of methemoglobinemia cases are diagnosed incidentally, and only 1.5% are detected through blood gas analysis using conventional carbon monoxide oximetry. As a result, many cases may go undetected.^[3]^

Foodborne methemoglobinemia is commonly associated with the ingestion of leafy vegetables in infants and young children, and its risk in this population is well recognized. However, to date, only a few cases of methemoglobinemia in adults resulting from an unbalanced dietary intake of leafy vegetables have been reported. In the current case, the patient experienced prolonged dizziness without a definitive diagnosis, which ultimately led to a fall and a resulting femoral fracture. We report this case to raise awareness of the potential for severe methemoglobinemia in adults due to dietary factors.

## 2. Case report

### 2.1. Patient information

A 60-year-old female experienced impaired mobility after a fall at home. She contacted the emergency services department and was initially admitted to the emergency room (ER) of another hospital. She had experienced lightheadedness for several years; however, no medical evaluation identified an apparent cause. She experienced chronic headaches and was diagnosed with trigeminal neuralgia, for which she regularly received tramadol hydrochloride and acetaminophen.

The results of a previous examination at another hospital revealed an elevated methemoglobin (MetHb) level on blood gas analysis, along with a left femoral fracture. The patient was transferred to our hospital for further treatment.

In her 40 seconds, she developed an eating disorder and modified her diet to consist solely of ~700 g of snapper sashimi and embryo bread, along with 700 g of cabbage, lettuce, and other leafy greens, accounting for 1200 kcal per day. She considered this adjusted and unbalanced diet to be “healthy” and maintained it daily.

The patient was 150-cm tall and weighed 45 kg at the time of admission, a significant reduction from her previous weight of 120 kg in her 20 seconds.

### 2.2. Clinical findings, timeline, and diagnostic assessment

Upon arrival at the ER of the previous hospital, the patient was conscious with a Glasgow Coma Scale score of 15 (E4V5M6). Her vital signs were as follows: blood pressure, 104/69 mm Hg; heart rate, 82 bpm; and respiratory rate, 20 breaths/min, with no signs of respiratory distress. Oxygen saturation was maintained at 80% on a reservoir mask (10 L of supplemental oxygen). Arterial blood gas analysis performed using a Radiometer ABL800 FLEX blood gas analyzer (Radiometer, Copenhagen, Denmark) revealed a pH of 7.451, partial pressure of carbon dioxide of 29.2 mm Hg, and partial pressure of oxygen of 244 mm Hg. The bicarbonate level was 20.1 mmol/L, lactate level was 0.7 mmol/L, arterial oxygen saturation was 95.5%, and the methemoglobin level was 25.0%.

**Figure 1.**
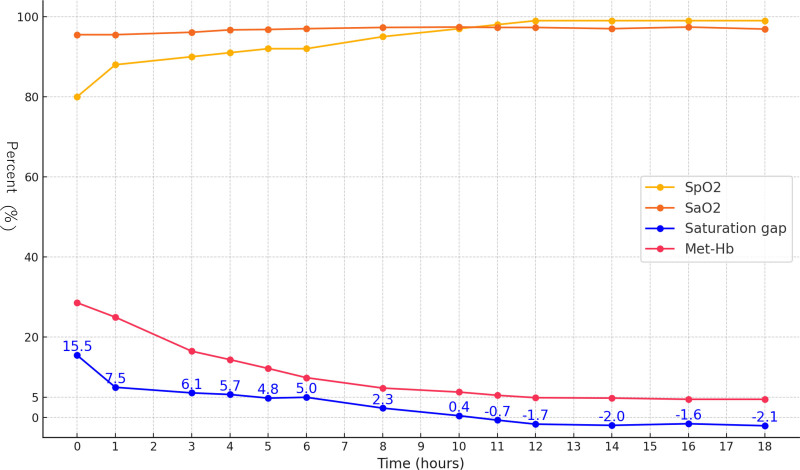
SpO_2_, SaO_2_, MetHb values, and saturation gap over time. MetHb = methemoglobin, SaO_2_ = arterial oxygen saturation, SpO₂ = Saturation of peripheral oxygen.

A saturation gap was observed (Fig. 1). The saturation gap refers to the difference between SpO₂ (saturation of peripheral oxygen) and SaO₂, typically defined as a discrepancy of ≥5%. In the current case, despite improved partial pressure of arterial oxygen levels with oxygen administration, SpO₂ remained persistently low, and the patient exhibited cyanosis. Since SpO₂ is determined via light absorption using pulse oximetry, it can be influenced by the presence of abnormal hemoglobin levels. Methemoglobinemia was identified as the underlying cause of the saturation gap. The discrepancy in the gap was resolved by normalization of the elevated methemoglobin levels.

Chest auscultation revealed no abnormalities; however, the patient developed cyanosis. Physical examination revealed external rotation and shortening of the left lower extremity, consistent with a left femoral neck fracture. The patient reported tenderness in the left hip. She had no history of diarrhea and was taking the Toaraset combination tablet, “Nippon-zoki.”^[4]^ She had no history of using groundwater as a source of drinking water.

Electrocardiographic findings on admission to the ER demonstrated a regular sinus rhythm without any ST segment or T wave abnormalities. The mean corrected QT interval was 343 ms. Physical examination revealed no systolic ejection murmurs during cardiac auscultation. No findings indicated aortic valve stenosis. Based on these clinical results, no evidence of cardiac syncope was noted. Additionally, the urine drug screening test (SygnifyER) performed at our hospital was negative for any drug reactions.

Laboratory tests on admission revealed a white blood cell count of 7600/μL (reference range: 3500–9000/μL), hemoglobin (Hb) level of 9.8 g/dL (12.0–16.0 g/dL), and mean corpuscular volume of 99.7 fL (80–100 fL). The platelet count was 17.1 × 10^4^/μL (15.0–35.0 × 10⁴/μL). Liver function tests revealed aspartate aminotransferase levels of 37 U/L (normal range, 13–30 U/L) and alanine aminotransferase levels of 40 U/L (normal range, 7–23 U/L). Total bilirubin was 0.67 mg/dL (0.2–1.2 mg/dL), lactate dehydrogenase was 272 U/L (124–222 U/L), and albumin was 3.2 g/dL (4.1–5.1 g/dL). Renal function tests revealed a blood urea nitrogen level of 11.6 mg/dL (8–20 mg/dL) and a creatinine level of 0.43 mg/dL (0.6–1.0 mg/dL). Electrolytes were within acceptable ranges, with sodium at 139 mEq/L (136–145 mEq/L), potassium at 3.0 mEq/L (3.5–5.0 mEq/L), and chloride at 111 mEq/L (98–107 mEq/L). The C-reactive protein level was low at 0.02 mg/dL (<0.30 mg/dL). The reference ranges were based on MedlinePlus^[5]^ (Table 1).

**Table 1:** Laboratory data representing the status of the patient at admission.

Complete blood count			
WBC	7600	/μL	(3500–9000)
Hb	9.8	g/dL	(12.0–16.0)
MCV	99.7	fL	(80–100)
PLT	17.1	10^4^/μL	(15.0–35.0 × 10^4^)
Serum biochemistry			
AST	37	U/L	(13–30)
ALT	40	U/L	(7–23)
T-Bil	0.67	mg/dL	(0.2–1.2)
LDH	272	U/L	(124–222)
Alb	3.2	g/dL	(4.1–5.1)
BUN	11.6	mg/dL	(8–20)
Cre	0.43	mg/dL	(0.6–1.0)
CRP	0.02	mg/dL	(<0.30)
Na	139	mEq/L	(136–145)
K	3.0	mEq/L	(3.5–5.0)
Cl	111	mEq/L	(98–107)

Alb = Albumin, ALT = alanine aminotransferase, AST = aspartate aminotransferase, BUN = blood urea nitrogen, Cre = creatinine, CRP = C-reactive protein, Hb = hemoglobin, MCV = mean corpuscular volume, PLT = platelets, T-Bil = total bilirubin, WBC = white blood cells.

### 2.3. Interventions

The MetHb level of the patient was 25% upon arrival at the ER of our hospital. Methylene blue, which is reduced by nicotinamide adenine dinucleotide phosphate, subsequently reduces MetHb and is thus sometimes used as a treatment for methemoglobinemia. However, administration of methylene blue (MB) is recommended when the MetHb concentration reaches 30% or higher in the blood.^[6]^ In the present case, we considered using MB; however, as the patient improved rapidly with oxygen therapy alone, MB was not administered; instead, we applied normobaric oxygen therapy without the administration of MB. This therapy consisted of 10 L/min via a reservoir mask for the initial 2 hours, followed by a high-flow nasal cannula (60 L/min, 100% oxygen) for 12 hours, and then 3 L/min via a standard nasal cannula for the subsequent 6 hours. Subsequently, the patient was maintained in ambient air. The clinical course of blood gas analysis is shown in Table S1, Supplemental Digital Content, https://links.lww.com/MD/R6.

### 2.4. Follow-up and outcomes

The MetHb level of the patient decreased to 4.5% at 16 hours after admission, which is within the acceptable normal range (typically < 5%).^[1]^ She was discharged from our hospital and transferred to another hospital for treatment of the left femoral neck fracture on the 6th day of hospitalization.

## 3. Discussion

According to research from Johns Hopkins University, 25% of methemoglobinemia cases are incidentally identified. Conventional blood gas analysis using carbon monoxide oximetry detected only 1.5% of cases with elevated MetHb levels. Accurate diagnosis requires advanced and expensive equipment along with invasive testing methods. Therefore, the actual number of methemoglobinemia cases may be underestimated, and many cases may go undiagnosed.^[3]^

The causes of methemoglobinemia are classified into 2 types: congenital and acquired.^[1]^ This case was considered acquired, as the patient had no prior symptoms suggestive of methemoglobinemia and no relevant family history. Most acquired methemoglobinemia cases are associated with the use of drugs or chemicals. Medications, such as dapsone, phenazopyridine, primaquine, sulfasalazine, sulfamethoxazole, and rasburicase, are known contributors. The use of underground water was considered a possible risk factor (Table 2). Additionally, dietary sources, including leafy vegetables, meat, and fish, can contain preservatives or colorants that contribute to foodborne methemoglobinemia.^[2]^

**Table 2:** Various causes of methemoglobinemia.

Category	Cause	Description
Congenital	Cytochrome b5 reductase deficiency	Autosomal recessive. TypeⅠ (RBC-specific) and Type Ⅱ (systemic).
	Hemoglobin M disease	α or β globin chain mutation stabilizing oxidized iron (Fe3^+^).
	G6PD/Pyruvate kinase deficiency	Impaired NADH or NADPH production reduces MetHb reduction.
Acquired	Local anesthetics (e.g., benzocaine)	Oxidative stress; FDA warnings on benzocaine.
	Medications (e.g., iNO treat, dapsone, sulfa drug)	Cause MetHb formation via oxidative mechanisms.
	Chemicals (e.g., aniline dyes, arsenic)	Occupational or pesticide-related exposure.
	Inhaled nitrite (recreational use)	Transient oxidative stress induction.
	Nitrates/nitrites (e.g., water, food)	In this case. May case “Blue baby syndrome” in infants.
	Special conditions (e.g., radiotherapy, chemotherapy)	Drug-induced, radiotherapy, or environmental exposure.

FDA = Food and Drug Administration, G6PD = glucose-6-phosphate dehydrogenase, iNO = inhaled nitric oxide, MetHb = methemoglobin, NADH = nicotinamide adenine dinucleotide (reduced form), NADPH = nicotinamide adenine dinucleotide phosphate (reduced form), RBC = red blood cell.

After reviewing the patient’s daily dietary habits, we observed that she had not taken any of the suspected medications or used groundwater in her daily routine. Additionally, she did not regularly consume meat or fish products containing preservatives or colorants that are known to cause methemoglobinemia. However, she reported a daily intake of ~700 g of leafy green vegetables, particularly lettuce and cabbage.

Based on a daily intake of ~350 g of lettuce and cabbage, the estimated total nitrate intake was calculated to be ~16.5 mg/kg/day. This estimation was based on a median nitrate concentration of 1886 mg/kg for lettuce and 223 mg/kg for cabbage, as reported by the European Food Safety Authority in a comprehensive analysis of nitrate levels in vegetables across the European Union^[7]^ and, using domestic Japanese monitoring data (fresh-weight nitrate concentrations of ~1100 mg/kg for lettuce and ~700 mg/kg for cabbage)^[8]^ and a patient body weight of 45 kg, the estimated exposure was 14.0 mg/kg/d. This value corresponded to an intake 4.4-fold higher than the daily tolerable intake level for nitrate of 3.7 mg/kg/d defined by the World Health Organization (Table 3). The actual nitrate content may vary depending on factors such as season, cultivation methods, and variety, and is also influenced by the nitrate content of the cultivation soil. Although the actual nitrate content may vary depending on factors such as season, cultivation method, and variety, the European Food Safety Authority-reported medians serve as reasonable estimates for risk assessment.

**Table 3:** Estimated total nitrate intake from leafy vegetables.

Region	Japan*	EU (EFSA)†
Nitrate intake in lettuce (mg/d)	385	660.1
Nitrate intake in cabbage (mg/d)	245	78.05
Total nitrate intake (mg/d)	630	738.15
Nitrate intake per body weight (mg/kg/d)	14.0	16.4
ADI (mg/kg/d)	3.7	3.7
Ratio of nitrate intake per body weight to ADI	3.78	4.46

ADI = acceptable daily intake, EFSA = European food safety authority.

* Using domestic Japanese monitoring data fresh-weight nitrate concentrations of ~1100 mg/kg for lettuce and ~700 mg/kg for cabbage.^[8]^

† This estimation was based on a median nitrate concentration of 1886 mg/kg for lettuce and 223 mg/kg for cabbage, as reported by the EFSA in a comprehensive analysis of nitrate levels in vegetables across the European Union (EU).^[7]^

**Figure 2.**
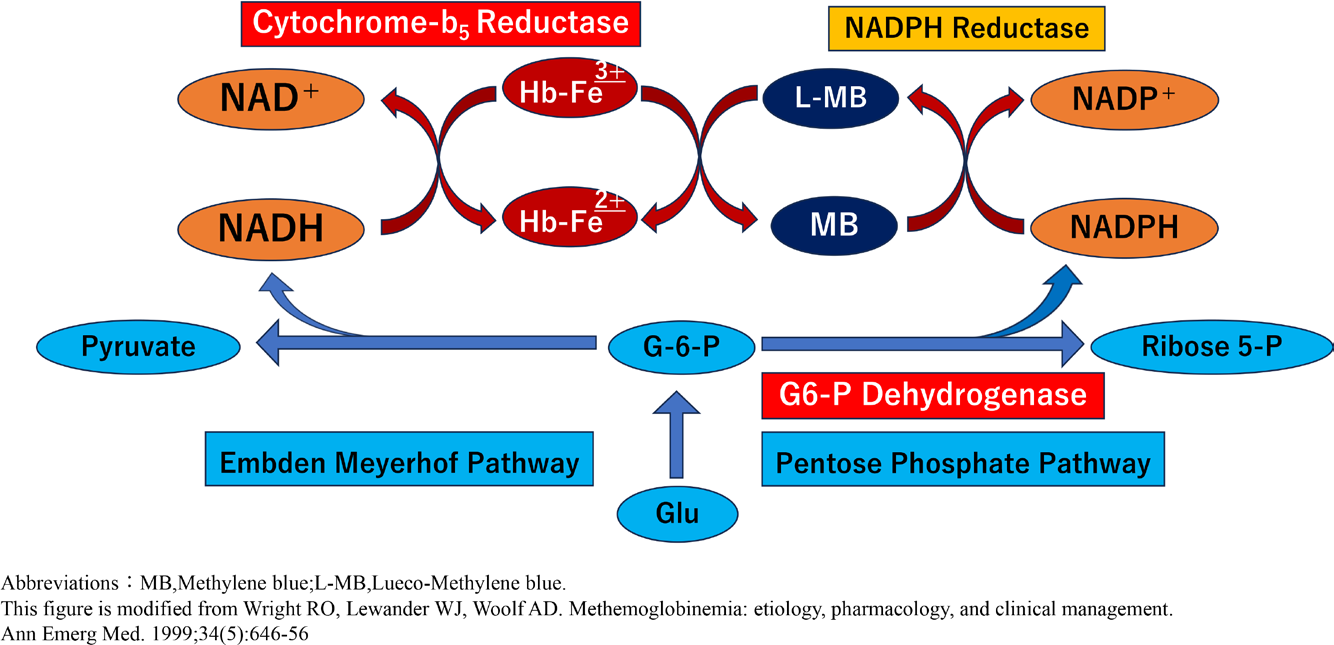
Endogenous enzymatic pathways of methemoglobin reduction and glycolysis. MB = methylene blue, L-MB = leuco-methylene blue (Adapted from Wright Ro et al^[7]^). This figure illustrates key endogenous systems that reduce methemoglobin to hemoglobin, including cytochrome b5 reductase and Nicotinamide Adenine Dinucleotide Phosphate (NADPH)-dependent pathways supported by glycolysis. Methylene blue enhances this reduction via the NADPH-dependent mechanism in clinical settings.

**Figure 3.**
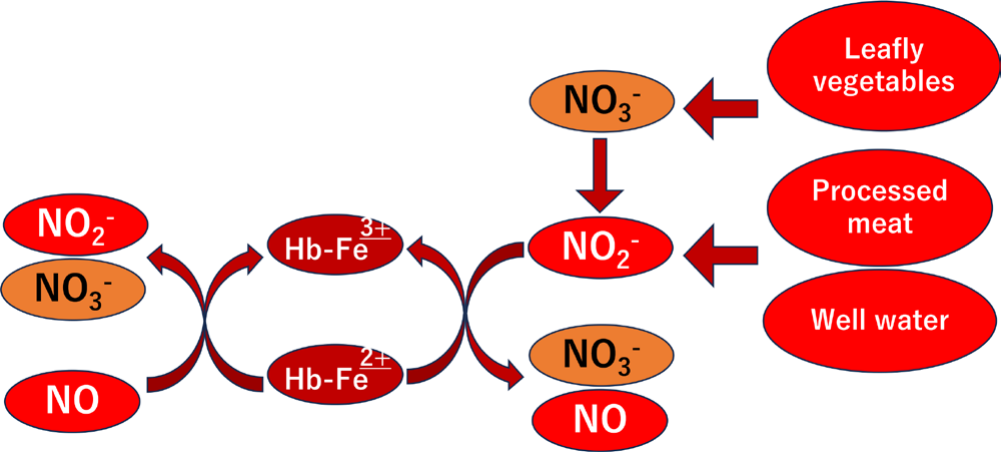
Exogenous pathways of methemoglobinemia from dietary sources. (adapted from Umbreit^[8]^). This figure depicts exogenous pathways of methemoglobinemia through nitrate and nitrite exposure from leafy vegetables, processed meats, and contaminated well water. These sources promote the oxidation of hemoglobin via nitrite accumulation following metabolic conversion.

Several studies have reported food-induced methemoglobinemia. A systematic review reported that most cases (568 patients) occurred in children and infants, with a median age of 6 years.^[2]^ Since food-induced methemoglobinemia is often associated with the ingestion of leafy green vegetables in children and infants, the risk of dietary methemoglobinemia in this population is well recognized. The pathophysiological mechanism underlying methemoglobinemia resulting from a high dietary intake of nitrate-rich leafy vegetables is illustrated in Figures 2 and 3.

Food-induced methemoglobinemia occurs easily in pediatric and infant cases. Infants under 3 months of age have limited gastric acid secretion, leading to altered gut flora. These conditions increase their sensitivity to the nitrate reduction reaction.^[2]^ Although the European Union has set a maximum acceptable daily intake of nitrates, no such standard currently exists in Japan. A systematic review attributed cases of diet-induced hyper methemoglobinemia in adults to the consumption of nitrate-rich foods, such as spoiled vegetable soups and sausages, that were prepared without considering the nitrate content.^[2,9]^

Additional examples of food-induced methemoglobinemia unrelated to eating disorders, particularly in children, include exposure to nitrate-contaminated well water and processed meat containing high levels of sodium nitrite preservatives. Shrock et al described a case of severe methemoglobinemia involving infant twins due to ingestion of sorghum syrup prepared with nitrate-rich well water.^[10]^ Theobald et al reported a father and daughter who developed methemoglobinemia after consuming home-cured beef jerky.^[11]^ Moreover, a recent report emphasized the ongoing risk of food and waterborne nitrate exposure by highlighting a cluster of neonatal methemoglobinemia cases caused by contaminated water in a maternity ward.^[12]^

Conversely, a few reports of methemoglobinemia in vegetarian adults have been presented.^[9]^ However, currently only a few reported cases, and the full extent of the risk is not well understood in this population. Thus, caution should be exercised in cases of eating disorders, as they can affect electrolyte and hormone secretion.^[9]^ In the present case, a medical history interview, which assessed the patient’s history of exposure to medications, chemicals, nitrite-containing foods, and well water, was conducted to confirm exposure; however, we did not conduct testing for congenital enzyme abnormalities or glucose-6-phosphate dehydrogenase deficiency. Furthermore, as normalization of methemoglobin levels following dietary guidance could not be tracked during outpatient follow-up, the possibility that hyper methemoglobinemia arose from other factors cannot be definitively ruled out. Furthermore, this case involved a proximal femoral fracture due to a fall, making it impossible to rule out the possibility that the patient’s anemia was acute, resulting from the fracture. Furthermore, as the patient’s baseline Hb value is unknown, we could not determine whether the anemia preceded and contributed to the symptoms or developed after the fracture. While dietary methemoglobinemia, as described above, was strongly suspected as the cause of the dizziness, this point also could not be definitively established.

## 4. Conclusion

The risk of food-induced methemoglobinemia in adults, particularly due to the excessive intake of leafy vegetables, is not well recognized. However, such dietary habits should be considered a potential cause of decreased SpO₂, and similar cases should be approached with caution. Therefore, detailed information on dietary intake is necessary.

## Acknowledgments

The authors wish to express their gratitude for the patient’s consent to use the data.

## Author contributions

**Conceptualization:** Katsuki Kono.

**Data curation:** Akihiro Noda.

**Investigation:** Katsuki Kono, Junko Yamaguchi.

**Supervision:** Kosaku Kinoshita.

**Visualization:** Katsuki Kono, Junko Yamaguchi.

**Writing – original draft:** Katsuki Kono.

**Writing – review & editing:** Katsuki Kono, Junko Yamaguchi.

## Supplementary Material


